# Assessment rate of true dorsogluteal intramuscular drug injection using ultrasonography

**DOI:** 10.12669/pjms.35.4.313

**Published:** 2019

**Authors:** Ozkan Ozen, Mucahit Gunaydin, Aptekin Tosun, Zafer Unsal Coskun, Kursad Aytekin, Selcuk Takir

**Affiliations:** 1 Ozkan Ozen, MD. Assistant Professor, Department of Radiology, Alanya Alaaddin Keykubat University, Antalya, Turkey. Faculty of Medicine, Debboy District, Giresun University, Giresun, Turkey; 2 Mucahit Gunaydin, MD. Assistant Professor, Department of Emergency Medicine, Faculty of Medicine, Debboy District, Giresun University, Giresun, Turkey; 3 Alptekin Tosun, MD. Associate Professor, Department of Radiology, Faculty of Medicine, Debboy District, Giresun University, Giresun, Turkey; 4 Prof. Zafer Unsal Coskun, MD. Department of Radiology, Istanbul Sultan Abdulhamid Han Education and Research Hospital, Istanbul, Turkey. Faculty of Medicine, Debboy District, Giresun University, Giresun, Turkey; 5 Kursad Aytekin, MD. Assistant Professor, Department of Orthopaedics and Traumatology, Faculty of Medicine, Debboy District, Giresun University, Giresun, Turkey; 6 Dr. Selcuk Takir, Associate Professor, Department of Pharmacology, Faculty of Medicine, Debboy District, Giresun University, Giresun, Turkey

**Keywords:** Intramuscular drug injection, Subcutaneous adipose tissue, Ultrasonography

## Abstract

**Objective:**

Medications are generally administered by either the enteric or parenteral route. With parenteral administration, intramuscular (IM) is the preferred approach because it increases the bioavailability of the drug, acts more quickly than the enteric route. The aim of this study was to determine the rate of true dorsogluteal intramuscular drug injection and to determine the causes for application failures in practice by ultrasonography (US).

**Methods:**

The study was conducted from May 1 to May 30, 2017 in Giresun University Education and Research Hospital, Giresun, Turkey. We examined 60 patients who were administered dorsogluteal IM injection with a 38.1mm length needle. After the injection, localization of medications (whether intramuscular or subcutaneous adipose tissue [SAT]) was evaluated by Ultrasound.

**Results:**

Female/male ratio of the patients was 27/33, with a mean age of 39.78±2.16 years. Obese/normal weight ratio was 20/40. The mean dorsogluteal area SAT thickness of obese and normal weight patients were 32.34±2.17 mm and 20.85±1.20 mm, respectively. In 23 of the patients, IM injected drug was observed in the SAT, while it was observed in the IM area in 37 patients. Medication was observed in IM area in 37 of 50 patients who dorsogluteal region SAT thickness was appropriate (SAT thickness lower than 33.1mm) for IM injection while it was seen in SAT area in 13 patients.

**Conclusions:**

SAT thickness values are important if IM drug injection is to be administered correctly. Unsuccessful IM injections may be seen even in patients with appropriate SAT thicknesses.

## INTRODUCTION

Medications are generally administered by either the enteric or parenteral route. With parenteral administration, intramuscular (IM) is the preferred approach because it increases the bioavailability of the drug, acts more quickly than the enteric route, and can be administered when enteric administration is impossible (e.g., in cases of vomiting, an uncooperative or unconscious patient, etc.).^1^,^2^ In this method of administration, drugs are often administered into the deltoid or gluteal muscles, which is the commonly selected region thanks to safety, ease of application, less pain compared to the deltoid site, and more volume of drug delivered.^1^,^3^ Medication injected into the gluteal region is absorbed almost completely and reaches the target site through the vena cava inferior without entering the enterohepatic cycle. However, this ideal description of administration may not always be possible in clinical practice. In the gluteal region, the thickness of adipose tissue varies throughout the person.^1^,^3^ For clinical IM applications, 1 to 1.5 inch (25–38 mm) IM injection needles are used, and the standard 32 mm IM injection needles reach a penetration depth of about 30 mm in clinical practice, penetrating into the muscle at least 5 mm to achieve a successful IM injection. This means that in patients with thick adipose tissue, it is likely that an injection will be administered into the subcutaneous adipose tissue (SAT) rather than IM.^1^,^4^

In our literature research, no similar previous studies were noted by ultrasonography regarding failure of IM injections derives from the patient’s SAT thickness or other reason. The aim of this study was to determine the proportion dorsogluteal IM injections that are indeed administered intramuscularly in clinical practice and to determine the causes for application failures in practice by ultrasonography.

## METHODS

The study was conducted from May 1 to May 30, 2017 after approval from the local ethics committee. Patients who underwent dorsogluteal IM injection of prescribed medications in the emergency room were included in the study. Inclusion criteria were as follows: patients older than 18 years, general condition suitable for ultrasonography (US) examination, and a signed consent to participate. Ultimately, 60 volunteers were recruited for the study. Verbal and written informed consent was obtained from all participants. A total of 14 nurses working in the emergency department administered the injections in the study. The least experienced nurse had five years’ experience and the most had 15 years of professional experience. The nurses who administered injections were blinded to the study by limiting of their work content and knowledge, because we thought that nurses could go out of routine practice. Nurses were only aware that a patient’s height, weight, and waist circumference would be measured. In the study, we collected no information about which patient was injected by which nurse. Injections for all patients were administered using a standard 1.5 inch (38.1 mm) 21 gauge needle. The ultrasound (US) examinations of the volunteers were performed in the emergency radiology department within 20 minutes of the injection, and all examinations were conducted by one radiologist (MD), who had 10 years of experience in US. The US examinations were performed using a real-time scanner (HI VISION Avius; Hitachi Medical Systems Co., Ltd., Tokyo, Japan) and a linear array transducer with a frequency of 5 to 13 MHz. The US examination evaluated the location of administered medication (whether IM or in SAT) and the SAT thickness at the dorsogluteal injection site. US evaluation was performed with the patient in the prone position, as with the injection procedure. SAT thicknesses were evaluated using US with the probe angled at 90 degrees on the skin, where the force applied on the probe was similar to that of the needle on the skin during injection (We received information about dorsogluteal IM injection from an experienced nurse before the study and follow up was by our service nurses). After the US examination, the weight, height, and waist circumference of the patients were measured. All the above-mentioned, as well as the sex of the patient, were recorded.

Data are presented as mean ± standard error of measurement, and n is the number of patients. Differences in tissue thickness in the dorsogluteal site between non-obese and obese patients in relation to body mass index (BMI) were analyzed using one-way ANOVA. Relationships between age and SAT thickness were assessed using correlation analysis (GraphPad Prism Version 5.00, GraphPad Software, San Diego, CA, USA), and a p value of less than 0.05 was considered statistically significant.

## RESULTS

The study’s participants included 27 male and 33 female patients with the mean age of 39.78 ± 2.16 years. The patients were classified into 1 of 2 groups according to BMI values: obese (n=20), those with a BMI higher than 25, and normal weight (n =40), those with a BMI lower than 25. In the obese group, the SAT thickness at the gluteal site and waist circumference were statistically higher than the normal weight group (p <0.05).

The sex, age, height, weight, waist circumference, BMI, dorsogluteal side of injection (left or right), and SAT thickness of the dorsogluteal injection site of all the patients are summarized in Table-I.

**Table I:** Sex, mean age, dorsogluteal injection side, height, weight, waist circumference, BMI, and subcutaneous fat tissue thickness at dorsogluteal injection site for all patients (both non-obese and obese groups).

Characteristics	All patients n = 60	Non-obese group n = 40	Obese group n = 20
Female	27	14	13
Male	33	26	7
Mean age	39.78 ± 2.16	34.65 ± 2.42*	50.05 ± 3.36
Dorsogluteal injection site: right side	13	4	6
Dorsogluteal injection site: left side	47	36	14
Height (cm)	167 ± 13.7	169 ± 15.8	161 ± 22.7
Weight (kg)	78.26 ± 2.06	72.63 ± 2.02	89.54 ± 3.59*
BMI	28.06 ± 0.68	24.72 ± 0.50	34.18 ± 0.69*
Waist circumference (cm)	94.12 ± 1.76	87.78 ± 1.77	106.80 ± 1.79*
Dorsogluteal site SAT thickness (mm)	24.68 ± 1.28	20.85 ± 1.20	32.34 ± 2.17*

SAT: subcutaneous adipose tissue

* p < 0.05 statistically significant differences compared to non-obese subjects (ANOVA).

**Fig. 1:**
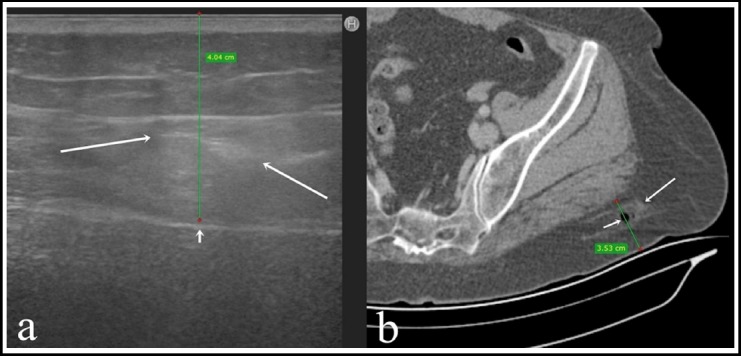
A 53-year-old female patient had diclofenac sodium injection. A) On US, dorsogluteal subcutaneous fat tissue (SAT) thickness was not appropriate for IM injection (green line:SAT thickness: 40.4 mm, short arrow: muscle fascia); drug is observed in SAT (long arrow). B) The patient also underwent CT scan immediately after the US examination. The CT scan revealed SAT injection densities (long arrow) and incorrectly injected air within these densities (short arrow). On CT, dorsogluteal SAT thickness was 3.53 mm, the body weight of patient lying in the supine position on the patient couch during CT may have altered the location and thickness of SAT (green line: SAT).

**Fig. 2:**
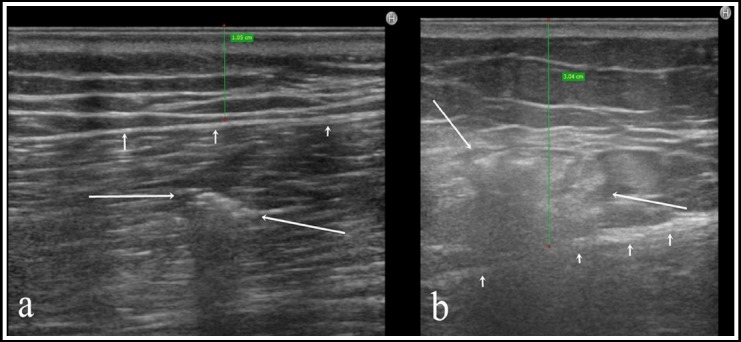
A 39-year-old male patient with a BMI of 24.7 who had a diclofenac sodium injection. A) On US, dorsogluteal subcutaneous fat tissue (SAT) thickness was appropriate for IM injection (green line: SAT thickness: 10.5 mm, short arrows: muscular fascia). Medication was observed in muscular tissue (long arrows). B) A 22-year-old female patient with a BMI of 2.58, who had a ceftriaxone injection. On US, dorsogluteal SAT thickness was appropriate for IM injection (green line: SAT thickness: 30.4 mm, short arrows: muscle fascia); the drug was observed in SAT (long arrows).

Of the 60 patients who received dorsogluteal IM injections, the US examination showed medication in the SAT in 23 (38.3%) patients and in the IM area in 37 (61.6%). The 60 patients in the study were then also divided into two groups depending on where the injections actually occurred: an IM injection group (patients with medication seen in the IM area, n=37) and the group who had injections in the SAT (patients with medication seen in the SAT, n=23). Statistical analysis of differences between the two groups revealed that the weight, BMI, waist circumference, and dorsogluteal SAT thickness were significantly higher in the SAT injection group than in the IM injection group (p <0.05). The values for the IM and SAT injection groups are shown in Table-II. Sample radiological images of patients who had IM and SAT injections are shown in Fig. 1 and 2.

**Table II:** Sex, average age, dorsogluteal side of injection administration, height, weight, waist circumference, BMI, and dorsogluteal subcutaneous fat tissue thicknesses of IM injection group (medication seen in IM area) and SAT injection group (medication seen in SAT).

Characteristics	IM injection group (n = 37)	SAT injection group (n = 23)
Female	12	8
Male	25	15
Mean age	39.22 ± 2.68	43.00 ± 4.41
Dorsogluteal injection site: right side	7	6
Dorsogluteal injection site: left side	30	17
Height (cm)	168.00 ± 16.6	1.65 ± 2.51
Weight (kg)	75.80 ± 2.30	85.25 ± 3.81*
BMI	26.79 ± 0.76	31.36 ± 1.17*
Waist circumference (cm)	92.22 ± 2.09	100.10 ± 2.98*
Dorsogluteal site SAT thickness (mm)	19.52 ± 1.04	32.81 ± 2.11*

SAT: subcutaneous adipose tissue

* Statistically significant difference compared to IM injection group, p < 0.05 (ANOVA).

## DISCUSSION

Clinically, it is difficult to evaluate whether injections are within the SAT or in the muscle.^1^ Muscule tissue has a richer vascular structure than SAT, so drugs injected into the muscle are absorbed better, and therefore, the bioavailability of drugs injected into muscle is better than with subcutaneous injections. Subcutaneous injections are preferred only for drugs that must enter the bloodstream slowly and constantly.^5^,^6^ This information suggest that the treatment efficacy of intra-fat injections is low, and it could thus be argued that injections made into the SAT result in a failure of treatment and an increase of treatment costs.

The different possible administration sites for injections include dorsogluteal, ventrogluteal, deltoid, rectus femoris, and vastus lateralis, but the most common site for injection in daily practice is the dorsogluteal area.^7^,^8^ In our hospital, unless obliged to do otherwise, nurses administer injections in this site, which is why injections administered in the dorsogluteal site were chosen for this study.

The first radiological study with IM injection was performed with computed tomography (CT).^9^ In the study of Burbridge^9^ fat thickness was measured in the gluteal area by CT. Burbridge found that subcutaneous fat thickness was thicker in females than in males.^9^ Further, according to our literature review, the first study on IM injections using US was carried out by Zaybak et al.^10^ In this study, dorsogluteal and ventrogluteal SAT thicknesses were measured in obese patients who had no injections. Their study revealed that an increase in BMI was proportionally related to the thickness of SAT in the dorsogluteal and ventrogluteal sites, and the authors had found that SAT in the ventrogluteal site is thicker when compared to the dorsogluteal region.^10^ Ours study’s findings are similar to these earlier studies in that dorsogluteal SAT was found to be significantly thicker in the obese group than in the non-obese group.

Chan et al.^1^ investigated whether dorsogluteal IM injections were truly IM using abdominal/pelvic CT. In that study, IM injection was performed with medication and 1 mL air prior to CT on patients prescribed IM injection by a physician. The authors evaluated 50 patients, 25 men and 25 women. Injection was performed by a single practitioner, who was aware of the study, using a 30-mm needle. They reported a successful IM injection rate of 80-90% in men with a dorsogluteal region SAT thickness up to 20 mm and in 100% of women. However, no successful IM injection was determined in male patients with SAT thicknesses exceeding 20 mm. Chan et al. may not have obtained accurate results since the body weight of patients lying in the supine position on the patient couch during CT may have altered the location and thickness of SAT.^1^ In our study, US evaluation was performed with the patient in the prone position, as with the injection procedure and practitioners were unaware of the study.

An interesting study of IM injections was published by Masuda et al.^11^ where the thickness of SAT in the gluteal area was measured using both ultrasound and “a near-infrared ray measuring device” called a PoccoStick. In this study, the measured values obtained with the PoccoStick device were thicker than the measurements obtained with ultrasound. Masuda et al.^11^ reported that development of a small device such as the PoccoStick, which can achieve more precise measurements, is required for practical use.

There are few complications in actual IM injections because of adequate blood circulation in muscle tissue, while injections administrated into SAT can cause various complications, some serious, due to the lack of drainage from fatty tissue. 1 SAT injection complications can include local irritation, pain, infection, neuropathy, hematomas, bleeding, persistent nodules (calcified granulomas), fibrosis, abscesses, tissue necrosis, gangrene, or muscle contraction.^1^,^12^

Taking into account that complications occur more frequently in SAT injections^1^, they in our study, where the length of the needle used for injection in all the patients was 1.5 inch (38.1 mm), the injected drug was located in SAT in 23 of the 60 subjects (38.3%). It has been reported that for a successful IM injection, the needle must penetrate into the muscle at least 5 mm.^4^ According to these guidelines, ours study; a successful IM injection should have been accomplished in all patients with a SAT thickness of 33.1 mm or less. However, the injected drug was found in SAT in 13 of 50 patients (21.6%) who dorsogluteal region SAT thickness - of 33.1 mm or less. The results indicate that obesity in patients and the associated thicker SAT might interfere with IM injections, although failures related to the practitioners cannot be overlooked. Nurse-induced failures may be due to improper positioning of the needle for IM injection.

### Limitations of the study

Nurses who administered the injections may have been informed about our study after 30 patients. In addition, the researchers did not know which nurse administered the injections to individual patients. Another limitation is that this study had a small number of participants in the obese patient group.

## CONCLUSION

It is important for practitioners to take into account the patient’s SAT thickness for the correct IM administration of the drugs and to perform injection correctly. The SAT thickness in patients to whom an IM injection is to be administered can be measured by US before injection, and practitioners who will perform injections could be given training on US evaluations of SAT prior to injection. However, the development of a practical device capable of accurately and easily measuring SAT thickness prior to IM injections may be the most appropriate solution overall.

### Authors’ Contribution

**OO, ST, KT, MG:** Conceived, designed and did statistical analysis & editing of manuscript.

**OO, AT, ZUC:** Did data collection and manuscript writing.

**OO, ST:** Did review and final approval of manuscript.

## References

[1] Chan VO, Colville J, Persaud T, Buckley O, Hamilton S, Torreggiani WC (2006). Intramuscular injections into the buttocks:are they truly intramuscular?. Eur J Radiol.

[2] Dayananda L, Belaval VV, Raina A, Chandana R (2014). Intended intramuscular gluteal injections:are they truly intramuscular?. J Postgrad Med.

[3] Tanioka T, Sakamaki S, Yasuhara Y, Tomotake M, Takase K, Watari C (2013). Optimal Needle Insertion Length for Intramuscular Injection of Risperidone Long-Acting Injectable (RLAI). Health.

[4] Larkin TA, Ashcroft E, Elgellaie A, Hickey BA (2017). Ventrogluteal versus dorsogluteal site selection:A cross-sectional study of muscle and subcutaneous fat thicknesses and an algorithm incorporating demographic and anthropometric data to predict injection outcome. Int J Nurs Stud.

[5] Kim H, Park H, Lee SJ (2017). Effective method for drug injection into subcutaneous tissue. Sci Rep.

[6] Nisbet AC (2006). Intramuscular gluteal injections in the increasingly obese population:Retrospective study. BMJ.

[7] Wynaden D, Tohotoa J, Al Omari O, Happell B, Heslop K, Barr L (2015). Administering intramuscular injections:how does research translate into practice over time in the mental health setting?. Nurse Educ Today.

[8] Ogston-Tuck S (2014). Intramuscular injection technique:an evidence-based approach. Nurs Stand.

[9] Burbridge BE (2007). Computed tomographic measurement of gluteal subcutaneous fat thickness in reference to failure of gluteal intramuscular injections. Can Assoc Radiol J.

[10] Zaybak A, Gunes UY, Tamsel S, Khorshid L, Eşer I (2007). Does obesity prevent the needle from reaching muscle in intramuscular injections?. J Adv Nurs.

[11] Masuda S, Tanioka T, Yasuhara Y, Atsuta A, Ito H, Motoki K (2016). Availability of Thickness Estimation of the Subcutaneous Fat by Using the Near infrared Ray Measuring Device. Int J Nurs Clin Pract.

[12] Cocoman A, Murray J (2008). Intramuscular injections:a review of best practice for mental health nurses. J Psychiatr Ment Health Nurs.

